# Cases presentation and relapse rates associated with specific risk factors

**DOI:** 10.1192/j.eurpsy.2024.116

**Published:** 2024-08-27

**Authors:** J. I. Mena Garcia, Relapse predictors of bipolar disorder

**Affiliations:** Psychiatry and Psychology Service, Hospital Clínic, Barcelona, Spain

## Abstract

**Abstract:**

This section will be destinated to the presentation of specific cases of patients with bipolar disorder admitted to our acute psychiatric ward. For each case, sociodemographic, clinical and environmental characteristics will be described and pharmacological treatment discussed. In addition, predictive and protective factors for mood relapses will be identified, and then, prospective information regarding their clinical prognosis will be provided in order to discuss with the attendees the impact of the mentioned factors on clinical outcomes.

**Disclosure of Interest:**

None Declared

